# Supplementary material for: Degradation of Four Major Mycotoxins by Eight Manganese Peroxidases in Presence of a Dicarboxylic Acid
Source: Toxins (Basel). 2019 Sep 27;11(10):566. doi: 10.3390/toxins11100566 (PMC6833064; doi:10.3390/toxins11100566)
Supplement: Supplementary file 1 [file toxins-11-00566-s001.pdf]

Supplementary Materials: Degradation of Four Major Mycotoxins by Eight Manganese Peroxidases in Presence of a Dicarboxylic Acid

Xiaolu Wang, Xing Qin, Zhenzhen Hao, Huiying Luo, Bin Yao and Xiaoyun Su

Table S1. Primers used in this study.

Primer	Sequence (5′ to 3′)	Usage	Gene (GenBank Accession Number)
IlMnP1-F	CGCGGATCCGCACCCTCTTCTAGAGTGACATGCAGT	Cloning of *IlMnP1*	*IlMnP1* (KX620478)
IlMnP1-R	TAAAGCGGCCGCTTACACAGGAACGATGGAGGTGGCG		
IlMnP2-F	CGCGGATCCGCAATCACCAAGCGTGTTGCTTGTCCT	Cloning of *IlMnP2*	*IlMnP2* (KX620479)
IlMnP2-R	CCGCTCGAGTTACGAGGGAGGGACAGGGGCGACAGA		
IlMnP4-F	CGCGGATCCGCTCCCCAAGACGTTACTGCCGC	Cloning of *IlMnP4*	*IlMnP4* (This article)
IlMnP4-R	CCGCTCGAGTTACGACGGAGGTACTGGAGGAATCG		
IlMnP5-F	CGGAATTCGCCGTCGTCAGGCGTGTCACTTG	Cloning of *IlMnP5*	*IlMnP5* (This article)
IlMnP5-R	CCGCTCGAGTTAGGACGGAGGGACAGGAGCGAC		
IlMnP6-F	CGGAATTCGCTATCACCAGACGTGTTGCGTGC	Cloning of *IlMnP6*	*IlMnP6* (This article)
IlMnP6-R	ATTTGCGGCCGCTTAAGACGGGGGAACAGGGGCAAC		
PcMnP1-F	GCTGAATTCGCAGTCTGTCCAGACG	Cloning of *PcMnP1*	*PcMnP1* (J04980.1)
PcMnP1-R	GCTGCGGCCGCGGCAGGGCCATC		
CsMnP-F	GCTCATATGGCACCGACCACCATTTGTCC	Cloning of *CsMnP*	*CsMnP* (MG190336.1)
CsMnP-R	GCTGGATCCTGCCGGACCATCAA		

The underlined sequences indicate restriction enzyme recognition sites.

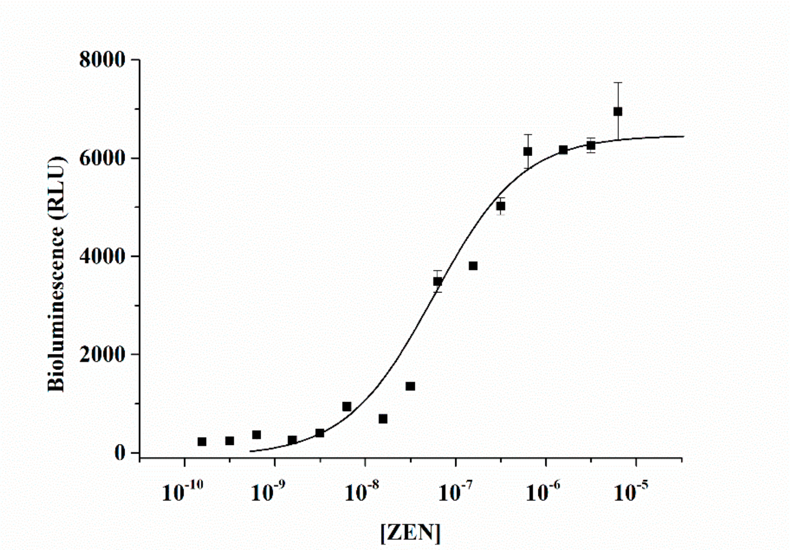

Figure S1. ZEN displays estrogenic activity on engineered *S. cerevisiae* BLYES. The BLYES strain was exposed to various concentrations of ZEN and the bioluminescent output was plotted against the corresponding concentration. X-axis (ZEN concentration) was on a log scale. The concentrations displayed were effective testing concentrations, i.e., the final concentrations after standard and BLYES reporter cells were mixed.

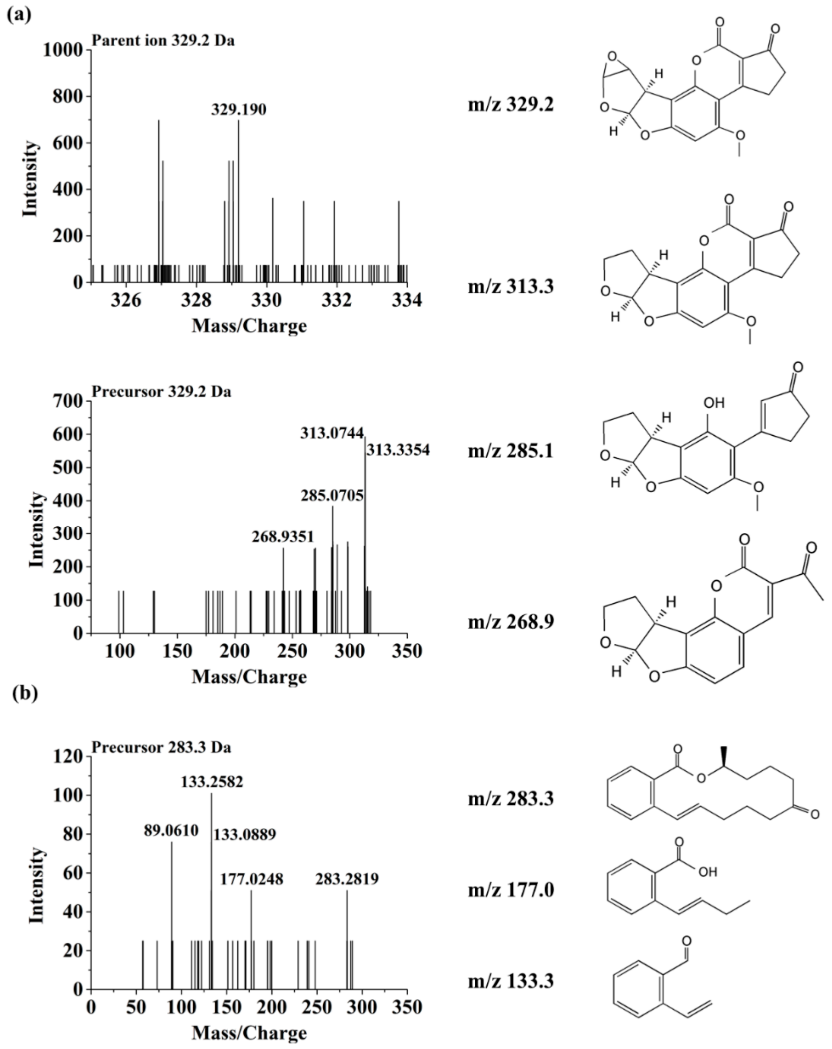

Figure S2. MS/MS spectrum of degradation products of AFB_1 and ZEN. (**a**) Degradation products of AFB_1 (Precursor 329.2 Da). (**b**) Degradation products of ZEN (Precursor 283.3 Da).

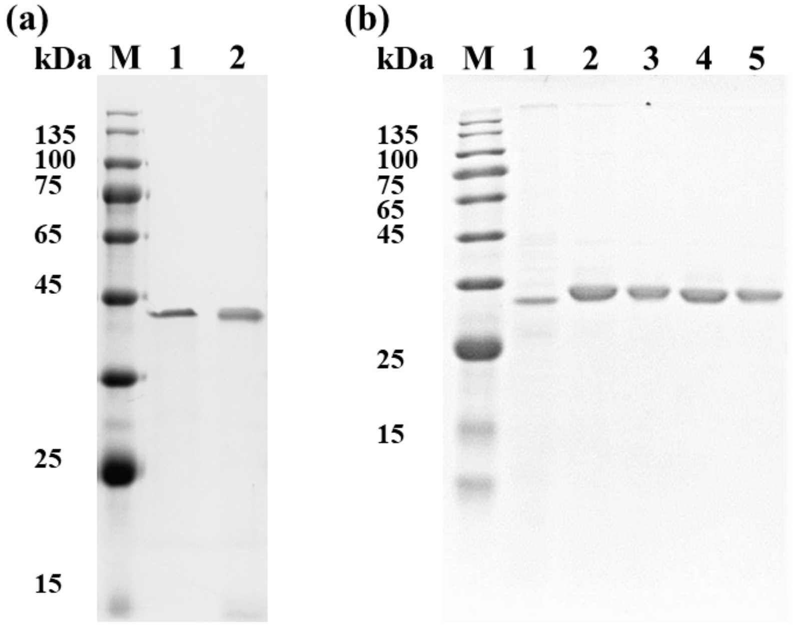

Figure S3. Purification of the recombinant MnPs. (**a**) Purification of *Cs*MnP and *PcMnP1*. Lane M, protein molecular mass marker; lane 1, *CsMnP*; lane 2, *PcMnP1*. (**b**) Purification of *IlMnP1*, *IlMnP2*, *IlMnP4*, *IlMnP5*, and *IlMnP6*. Lane M, protein molecular mass marker; lane 1, *IlMnP1*; lane 2, *IlMnP2*; lane 3, *IlMnP4*; lane 4, *IlMnP5*; lane 5, *IlMnP6*.

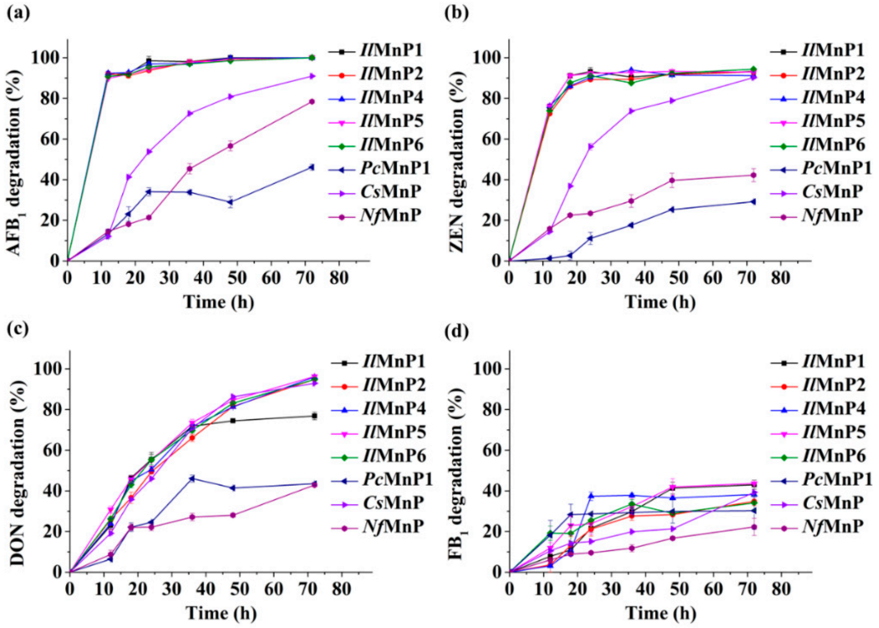

Figure S4. Time course analysis of degradation of four major mycotoxins by the eight MnPs. (**a**) AFB_1 degradation. (**b**) ZEN degradation. (**c**) DON degradation. (**d**) FB_1 degradation.

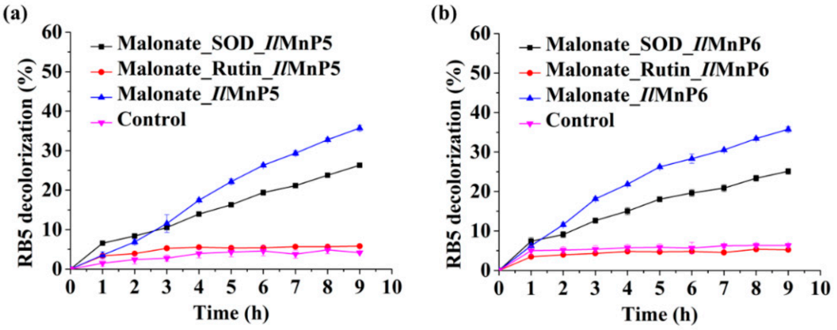

Figure S5. SOD and rutin suppressed RB5 decolorization with *IlMnP5* (**a**) and *IlMnP6* (**b**) as two model enzymes.

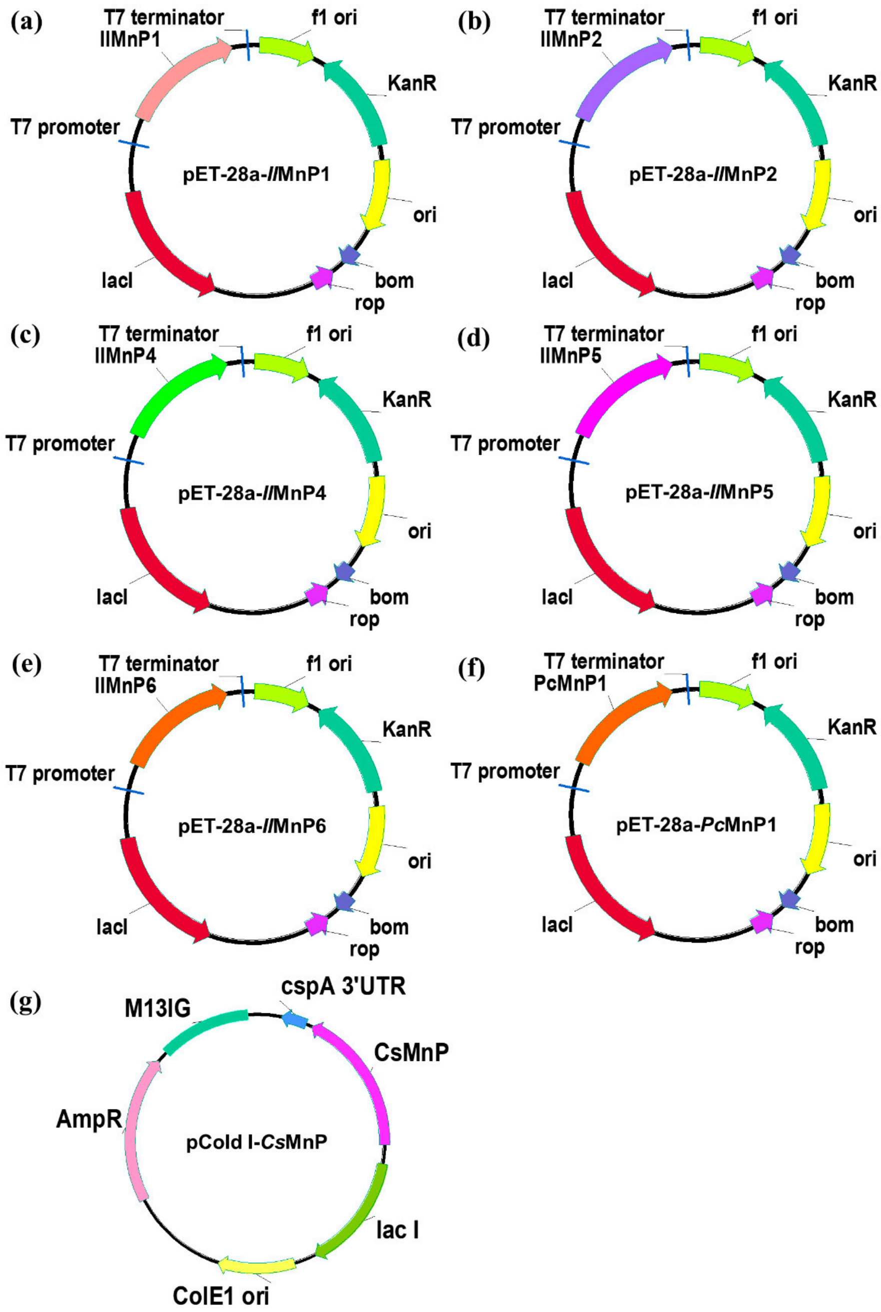

Figure S6. Schematic drawing of recombinant plasmids for expressing MnPs. (**a**) pET-28a-*Il*MnP1. (**b**) pET-28a-*Il*MnP2. (**c**) pET-28a-*Il*MnP4. (**d**) pET-28a-*Il*MnP5. (**e**) pET-28a-*Il*MnP6. (**f**) pET-28a-*Pc*MnP1. (**g**) pCold I-*Cs*MnP.
